# Yellow Fever

**Published:** 1873-11

**Authors:** Joseph Jones


*NEW YORK MEDICAL JOURNAL.*

Yellow Fever.—*Joseph Jones, M.D.*—The stomach contained one pint of black vomit, of a dark, purplish color, resembling in all respects defibrinated and altered blood. Under the microscope, the black vomit was found to contain numerous colored blood-corpuscles, variously altered, also cells from the mucous membrane of the stomach, and also broken capillaries.

This specimen of black vomit, taken from the stomach, twelve hours after death, contained some fibres, and the sporules and thallus of a plant, resembling the yeast-plant.

It is not probable that, *per se*, that is, in and of themselves, in their natural and uncomplicated and uncontaminated state, as developed in innocuous fluids, these low organisms are the cause of yellow fever; but they may become active agents in the dissemination of the peculiar *animal poison* which we believe to be the veritable *cause* of the disease.

All the facts in this complicated problem appear to be explained by the theory that the disease is produced by animal poison or product of the human body under certain circumstances, which has defined chemical relations to the blood and organs, inducing a distinct cycle of changes, and defined chemical and physical alterations, of the solids and fluids, which are as permanent and lasting as those resulting from the action of the small-pox poison.

According to this view, the poison of yellow fever may be directly communicated, or it may be communicated by the agency of simple plants and animalculæ; and not one but many species of these simpler forms of vegetable and animal organisms may become active agents in the spread of the disease.

